# Genetic and Neuroimaging Approaches to Understanding Post-Traumatic Stress Disorder

**DOI:** 10.3390/ijms21124503

**Published:** 2020-06-24

**Authors:** Sabah Nisar, Ajaz A. Bhat, Sheema Hashem, Najeeb Syed, Santosh K. Yadav, Shahab Uddin, Khalid Fakhro, Puneet Bagga, Paul Thompson, Ravinder Reddy, Michael P. Frenneaux, Mohammad Haris

**Affiliations:** 1 Functional and Molecular Imaging Department, Research Branch, Sidra Medicine, Doha 26999, Qatar; snisar1@sidra.org (S.N.); abhat@sidra.org (A.A.B.); shashem@sidra.org (S.H.); nsyed@sidra.org (N.S.); syadav@sidra.org (S.K.Y.); 2 Translational Research Institute, Hamad Medical Corporation, Doha 3050, Qatar; SKhan34@hamad.qa; 3 Department of Human Genetics, Sidra Medicine, Doha 26999, Qatar; kfakhro@sidra.org; 4 Department of Genetic Medicine, Weill Cornell Medical College, Doha 24144, Qatar; 5 Center for Magnetic Resonance and Optical Imaging, Department of Radiology, Perelman School of Medicine, University of Pennsylvania, Philadelphia, PA 19104, USA; puneetb@pennmedicine.upenn.edu (P.B.); krr@pennmedicine.upenn.edu (R.R.); 6 Imaging Genetics Center, Stevens Institute for Neuroimaging & Informatics, Keck USC School of Medicine, Los Angeles, CA 90033, USA; pthomp@usc.edu; 7 Academic Health System, Hamad Medical Corporation, Doha 3050, Qatar; mfrenneaux@hamad.qa; 8 Laboratory Animal Research Center, Qatar University, Doha 2713, Qatar

**Keywords:** PTSD, neuroimaging, PET, MRI, imaging genetics

## Abstract

Post-traumatic stress disorder (PTSD) is a highly disabling condition, increasingly recognized as both a disorder of mental health and social burden, but also as an anxiety disorder characterized by fear, stress, and negative alterations in mood. PTSD is associated with structural, metabolic, and molecular changes in several brain regions and the neural circuitry. Brain areas implicated in the traumatic stress response include the amygdala, hippocampus, and prefrontal cortex, which play an essential role in memory function. Abnormalities in these brain areas are hypothesized to underlie symptoms of PTSD and other stress-related psychiatric disorders. Conventional methods of studying PTSD have proven to be insufficient for diagnosis, measurement of treatment efficacy, and monitoring disease progression, and currently, there is no diagnostic biomarker available for PTSD. A deep understanding of cutting-edge neuroimaging genetic approaches is necessary for the development of novel therapeutics and biomarkers to better diagnose and treat the disorder. A current goal is to understand the gene pathways that are associated with PTSD, and how those genes act on the fear/stress circuitry to mediate risk vs. resilience for PTSD. This review article explains the rationale and practical utility of neuroimaging genetics in PTSD and how the resulting information can aid the diagnosis and clinical management of patients with PTSD.

## 1. Background

**Figure 1:**
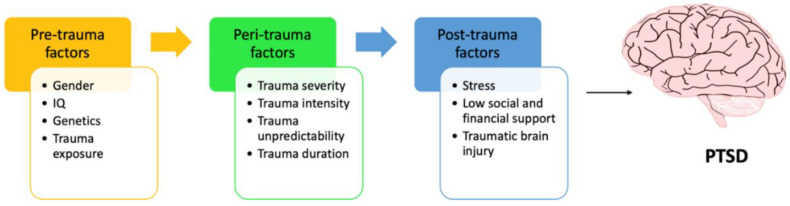
Trauma factors associated with post-traumatic stress disorder (PTSD). Pre-trauma factors include gender, IQ, family history, and various neurobiological factors while peri-trauma factors include the type and duration of trauma exposure. Finally, post-trauma factors include social support factors, coping skills, and cognitive flexibility.

Post-traumatic stress disorder (PTSD) is a psychological illness that can arise after a person experiences one or more traumatic events such as military combat, physical trauma due to injury, or other accidents such as domestic violence, rape, childhood trauma, neglect, or abuse. The disorder is characterized by feelings of panic, anxiety, fearfulness, and negative alterations in mood. Symptoms include distress and panic attacks, and the involuntary re-experiencing of the trauma during flashbacks or when exposed to memory triggers. There may also be disturbances in mood or impulse control, including aggressiveness, or exaggerated intensities of certain behaviors [1]. Furthermore, PTSD is a crucial area of research in psychiatry, to better understand how trauma exposure interacts with other factors in the development of PTSD. Certain risk factors can determine the development of PTSD in an individual, as not all individuals develop PTSD after trauma exposure. These risk factors can be classified as pre-trauma, peri-trauma, and post-trauma factors (Figure 1). Pre-trauma factors include gender, IQ, family history, and various neurobiological factors, while peri-trauma factors include the type and duration of trauma exposure. Finally, post-trauma factors include social support factors, coping skills, and cognitive flexibility [2]. 

Many neuroimaging studies have been performed to study the pathophysiology of PTSD by evaluating the associated structural, functional, and molecular changes in the brain. In the last few years, non-invasive imaging techniques have become more advanced, and neuroimaging has become accepted as a reliable and patient-friendly technique that can aid in the diagnosis of many neurological disorders. Many neural networks and pathways that play a role in PTSD have been uncovered, and these pathways can be studied in-depth due to the advances taking place in techniques for functional neuroimaging. The most common methods of imaging that are used in the study of PTSD are magnetic resonance imaging (MRI), positron emission tomography (PET), and proton (^1^H) magnetic resonance spectroscopy (PMRS) [3].

**Figure 2:**
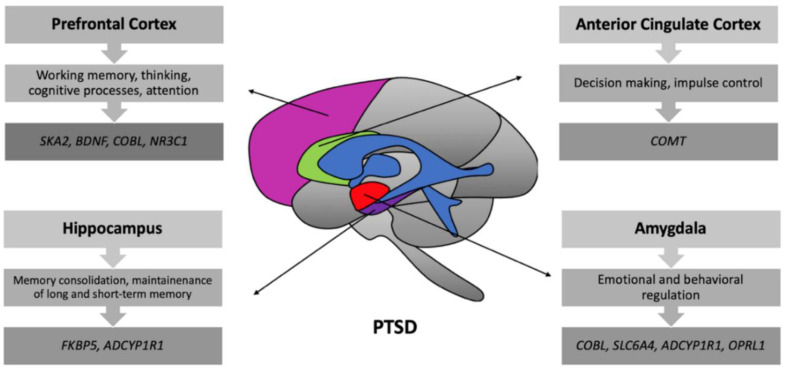
Genes associated with different brain regions in PTSD. In PTSD, genes such as *SKA2*, *BDNF*, *COBL*, and *NR3C1* are found to be associated with changes in the prefrontal cortex which is mainly involved in working memory processes. *COMT* is associated with changes in the anterior cingulate cortex (ACC) involved in the process of decision-making. *FKBP5* and *ADCYPIR1* are identified as being related to the hippocampal region, which plays an essential role in the maintenance of memory. Genes such as *COBL*, *SLC6A4*, *ADCYPIR1*, and *OPRL1* were associated with the amygdala that plays a vital role in the processes of emotional regulation.

The advances in imaging and genetic mapping have given birth to the field of imaging genetics, specifically in clinical and translational research. The combination of neuroimaging with genetic (or genomic) information can help to investigate the association between the various neuroimaging phenotypes with genetic markers. The presence of PTSD has been associated with a range of neuroimaging abnormalities, and people with PTSD also show subtle differences, on average, in the architecture of common genetic variants or single nucleotide polymorphisms (SNPs) in the genome. The genetic modifications contribute to the structural and functional changes in the brain of PTSD patients (Figure 2). Further links between these features can be explored by analyzing and testing the association between brain imaging techniques and genetic markers that are different, on average, in individuals with PTSD compared to normal healthy controls. This combination of imaging with genetics also shows promise in understanding dysregulated genes pathways that affect the neural systems that control stress and processing of fear, which underlie PTSD. In genetic studies of PTSD and related psychiatric disorders, many new developments include analysis of gene expression, copy number variants, and sequencing of the whole genome. These approaches, used on their own or in combination with imaging, can be useful for the identification and investigation of the genetic pathways involved in PTSD.

This review article provides detailed information on neuroimaging and genetic differences found in people with PTSD and how this information can be used to assist the early diagnosis and clinical management of PTSD patients.

## 2. Neuroimaging in PTSD 

MRI has been used to diagnose many neurological disorders and to investigate standard features observed in psychiatric illnesses, as well as pathologic mechanisms underlying these illnesses. MRI provides a whole-brain analysis of specific brain structures, including data on the cortical and subcortical structure volumes in patients who have PTSD. Useful diagnostic information on brain systems involved in emotional processing, or responsible for problems with memory and attention, is provided by both task-based and resting-state fMRI. MRI is also sensitive to structural and metabolic changes in the brain, offering measures that can help in detecting the early onset of PTSD. Neuroimaging studies in PTSD have been summarized in (Table 1).

### 2.1. Structural Brain Changes 

In addition to morphological characteristics and the anatomical location of the organ, T1, and T2 MR signals (T1 and T2 measures are relaxometric parameters that relate to the decay of the MRI signal; they depend on the type of tissue and are sensitive to pathological processes such as stroke, edema, demyelination, or neuronal atrophy) offer a crucial source of information in the explanation of pathological processes [14]. For example, studies have shown lower volumes of the amygdala and hippocampus, on average, in patients affected with PTSD as compared to non-PTSD patients or healthy controls. One such international study, by the ENIGMA Consortium and Psychiatric Genetics Consortium, analyzed neuroimaging and clinical data from 1868 subjects (794 PTSD patients) comprising 16 unique cohorts, representing the most extensive neuroimaging study of PTSD to date. In a meta-analysis of all samples, hippocampal and amygdala volumes were significantly reduced in subjects with PTSD compared to trauma-exposed control subjects [15]. Also, there has been a volumetric reduction seen in the CA3 (a subfield of the hippocampus) and dentate gyrus (a subfield of the hippocampus containing neural stem cells) [16]. These findings suggest that the volume of the hippocampus might be a risk factor for PTSD development. However, it is not fully understood whether the trauma solely causes the volume reduction seen in patients or if a pre-existing lower volume increases vulnerability to develop PTSD. Lower anterior cingulate cortex (ACC), rostral anterior cingulate cortex (rACC) volumes, and decreased gray matter densities have also been found in combat veterans suffering from PTSD [17]. Reduced volumes of the rostral ventromedial prefrontal cortex [18,19,20] and reduced grey matter in the ACC, medial frontal cortex, middle and superior frontal gyrus, paracingulate gyrus, and precuneus cortex has been observed in individuals with PTSD compared to trauma-exposed controls [21]. It has been reported that the hippocampus volume in PTSD patients may increase after treatment with paroxetine, which is a serotonin reuptake inhibitor [22]. Another study analyzed the differences in cortical thickness in patients who had developed PTSD resulting from a mining disaster to those of individuals without PTSD but were survivors of the coal mine disaster. They found cortical thinning in the regions of the right inferior and parahippocampal gyrus and left parietal lobe in individuals with PTSD compared to controls [23]. A further study showed that the co-twins of combat exposed veterans with PTSD had a higher risk of developing PTSD themselves. The study states that smaller hippocampi in PTSD represent a pre-existing, familial vulnerability factor rather than the exposure to trauma. In addition, the co-twin of combat exposed veterans without PTSD had a lower risk of developing PTSD, and the study mentioned that in the case of non-identical twins, the effect of both heredity and environment has to be considered.

Additionally, the study found no differences in the hippocampal volumes of non-identical twins [24]. A negative correlation with the Clinician-Administered PTSD scale (CAPS) score was found with the cortical thickness of the right frontal gyrus region in PTSD patients, which suggests that the cortical thinning may be linked with the severity of the symptoms [25]. Another study based on a surface-based morphometry (SBM) method observed lower cortical thickness in parietal, frontal and occipital lobes, hippocampus, and cingulate cortex and higher cortical thickness in the calcarine cortex in patients with PTSD. On average, there was a 10.75% and 9.09% cortical thickness reduction in the volume of the hippocampus and cingulate cortex of PTSD patients. The study thus concluded that the cortical thinning might be increased by stress, and this effect of stress appears to be higher in patients with PTSD compared to the non-PTSD control group [26].

Diffusion tensor imaging (DTI) studies found that microstructural degradation of the cingulum is associated with PTSD severity [27]. The cingulum bundle is an interconnecting pathway of neurons that interconnects multiple regions of the brain with the components of the limbic system—specifically with the hippocampus and anterior cingulate cortex. One study using DTI reported that white matter regions in the areas of the ACC, angular and precentral gyrus, and prefrontal cortex (PFC) of subjects with PTSD showed lower fractional anisotropy (FA) [28]. In another DTI study, FA was lower in the corpus callosum of adult patients and children with PTSD, which may reflect reduced interhemispheric connectivity within the corpus callosum [29]. The corpus callosum connects the two brain hemispheres and plays a significant role in working memory capacity and almost all cognitive functions of the brain. Another study comparing PTSD patients to controls without PTSD showed an increased FA with altered axial and radial diffusivity in the white matter region in PTSD patients [27]. In a murine imaging study, fear-conditioned mice that are prone to develop PTSD exhibited greater axial diffusivity changes than the mean diffusivity (MD) changes, relative to control mice. This finding was contrary to several previous brain plasticity DTI studies that reported higher MD and FA in PTSD in the gray matter regions of the brain [30]. Another DTI study reported a disequilibrium between the salience mode network (SMN) that includes the insula, putamen, and thalamus and the default mode network (DMN), which consists of the precuneus and angular gyrus in traumatized children with PTSD as compared to a group of traumatized controls without PTSD [31]. Multiple studies have shown MD changes, with higher mean values in the gray and white matter regions in PTSD [32].

### 2.2. Functional Brain Changes

Resting-state fMRI (rs-fMRI) is now a widely used approach to investigate connectivity within the brain and is performed when the subject is resting—defined as not completing a cognitive task or responding to visual or other sensory stimuli. One study conducted using rs-fMRI showed higher ALFF (amplitude of low-frequency fluctuation) values in patients with PTSD [33]. Another rs-fMRI study examined Regional Homogeneity (ReHo)—a measurement of the coherence of rs-fMRI signals—and showed higher coherence levels in the amygdala, hippocampus, putamen, and thalamus in patients with PTSD [34]. Another rs-fMRI study showed enhanced functional connectivity between the insula and amygdala in individuals with PTSD. These studies jointly imply that the expression of fear, which is a hallmark of PTSD, is linked with the activity and functional connectivity of the amygdala [35]. Similar studies involving combat veterans with and without PTSD and normal healthy controls found lower connectivity in the lateral prefrontal, para-hippocampal, and visual cortex regions in veterans with PTSD, perhaps implying there is a dissociation between sensory memory representations in PTSD [36]. Also, compared to healthy controls, veterans without PTSD showed hyper-connectivity in some brain regions, such as the insula, and hypo-connectivity in the precuneus region—a region responsible for the retrieval of memories of traumatic events [36]. A resting-state fMRI study conducted in individuals with PTSD, as well as trauma-exposed and non-trauma exposed individuals, showed higher activity in the insula and cerebellum regions in PTSD patients as compared to a non-trauma exposed group, and higher activity in the ventral prefrontal cortex as compared to trauma-exposed individuals. Meanwhile, lower activity was seen in the dorsal medial prefrontal cortex in individuals with PTSD, as compared to non-trauma exposed controls [37]

On the other hand, task-based fMRI is a functional MRI based technique that involves specific tasks. Task-based fMRI is used to discover Spatio-temporal patterns of blood flow in the brain that are elicited by neuronal activation when patients are asked to perform a short task or are challenged by a stimulus designed to elicit a response from the patients. This technique also helps to identify the more or less activated areas of the brain and can be used to evaluate and map patterns of correlation between perception of a stimulus, or performance of a task, and brain activation during the task. The strength of neural activation typically depends on task parameters, such as the level of pain, excitement or fear, and subject parameters, such as the person’s age, sex, and type of disorder. An emotional Stroop task study conducted in sexually-abused women with PTSD disorder and abused women without PTSD found that the ACC did not activate in response to these tasks. A reduction was seen in the functioning of the parietal cortex and visual association when women with PTSD were asked to name the color of an emotionally sensitive word such as rape. Another Stroop task-based study using fMRI found that in response to emotional/positive stimuli, there was increased activation of functional connectivity in the right amygdala, lateral, frontal, parietal cortices, and frontal and dorsal anterior cingulate cortex, and also in the left temporal cortex in subjects with sub-threshold PTSD symptoms [38]. Another Stroop task fMRI study was conducted among unmedicated individuals with PTSD and healthy controls. Results revealed disruption in the regions of the inferior and superior frontal and parietal cortices in individuals with PTSD. This suggests that in PTSD individuals, heightened emotional responses may interfere with the recruitment of brain regions associated with attention control [39]. An fMRI study using an oddball emotional task was performed on combat veterans with PTSD symptoms, showing increased neural activity in the ventral and dorsal limbic regions related to emotional stimuli with altered anterior cingulate function, which may be an indication of attentional bias in PTSD [40]. Another fMRI study using an oddball auditory task showed that individuals with PTSD had greater activation in the dorsal anterior cingulate cortex, left amygdala, and parietal somatosensory regions compared to matched normal healthy controls [41]. An fMRI study using a 3-back and identity task was conducted in women with PTSD who had been abused sexually; this study showed more significant deactivation of the posterior mid-parietal regions and greater activation of the left frontal regions than in healthy controls [42]. Another N-back task study revealed that the working memory of individuals with PTSD was found to be weaker than that of healthy individuals [43]. 

In fMRI studies based on emotional response tasks, the subject is presented with emotionally charged stimuli that may be visual, such as images with different moods and emotions or auditory stimuli, such as different sounds, or other sensory stimuli, such as different tastes or smells. Studies have shown differences in functional response related to stimuli, such as the presentation of words in the emotional category in patients who have PTSD [44,45,46]. Another fMRI study, involving an emotional processing task unrelated to trauma, included pictures that were categorized as positive, negative, or neutral. These pictures were shown to war veterans having PTSD, and patterns of brain activation were compared to those in war veteran controls without PTSD. In response to harmful stimuli, increased activation of the amygdala was seen in the ‘persistent’ patients (patients for whom PTSD persisted after months of treatment) compared to remitted patients (patients in remission) studied before treatment. When studied after treatment, in response to harmful stimuli, an elevated response of the anterior cingulate cortex and insula was seen in persistent patients versus remitted patients. Significant predictors found in the study that were involved in the persistence of PTSD were the activation of the dorsal anterior cingulate cortex, insula, and amygdala before treatment, in response to negative stimuli [47]. An fMRI study using the Go/No-Go inhibition task was conducted in patients with PTSD and healthy controls, and showed increased activation in the lateral frontal cortex, somatosensory and striatal regions, and decreased activation in the right cortical regions in patients with PTSD. These results suggest that the inhibitory control networks involved in attention control and working memory in PTSD may be compromised by the consistent activation of the right frontal cortex [48]. Another fMRI study showed lower activation in the rACC region in PTSD patients during a Go/No-Go task [49]. 

## 3. Genetic Differences in PTSD

**Figure 3:**
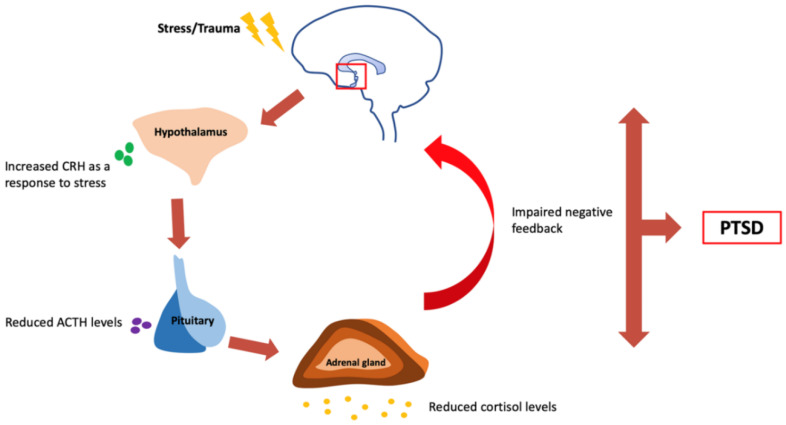
Dysregulation of the hypothalamic-pituitary-adrenal (HPA) axis in PTSD. An increase in corticotropin-releasing hormone (CRH) as a response to stress leads to a rise in the adrenocorticotropic hormone (ACTH) and cortisol levels, causing negative feedback to the hypothalamus to inhibit further release of CRH and ACTH. In PTSD, the ACTH responses are blunted, resulting in low cortisol secretion which does not send a signal to the hypothalamus to suppress stress hormone secretion resulting in impaired negative feedback.

The stress response systems in PTSD are found to be dysregulated, including disruptions to the hypothalamic-pituitary-adrenal (HPA) axis. Usually, stress or trauma stimulates the release of corticotropin releasing hormone (CRH) from the hypothalamus that, in turn, stimulates the pituitary to secrete adrenocorticotropic hormone (ACTH). Due to increased levels of CRH and ACTH, the adrenal gland secretes cortisol that feedbacks negatively to the hypothalamus to inhibit the release of ACTH and CRH further and initiate the fight and flight response. However, in the case of PTSD, there is blunted ACTH responses leading to reduced cortisol secretion and impaired negative feedback to the hypothalamus (Figure 3) [50]. This stress dysregulation is both physiological and psychological. Single nucleotide polymorphism in the glucocorticoid receptor gene can increase the sensitivity of the glucocorticoid receptor (GR) to cortisol, resulting in enhanced cortisol response [51]. One specific gene—*FKBP5*—alters the sensitivity of GR, and is considered to be an essential stress system regulator [52]. *FKBP5* is a candidate gene that may be considered as an underlying component of PTSD, and it has also been associated with peritraumatic dissociation—and perhaps even with risk for PTSD—in children who are medically injured [53]. Although no main effects directly relating to PTSD were found associated with the *FKBP5* polymorphism, some data suggest that the regulation of the amygdala related to the hypothalamic-pituitary-adrenal (HPA) axis stress-related genes can be altered after exposure to trauma during a critical period, and these early developmental alterations may then increase the risk of developing PTSD [49].

Some studies have also shown a link between PTSD and neuropeptide Y, also called NPY [54]. NPY plays a direct role in the reduction of fear; it may be a risk factor for anxiety disorders. On the other hand, monoamine oxidase B (*MAOB*) has an essential role in PTSD and may be a biomarker for various psychiatric illnesses. It is involved in the catabolism of tyramine, dopamine, and other monoamines that are involved in neural systems subserving attention. Polymorphisms in *MAOB* intron 13 and in the GABAA receptor subunit alpha 2 (*GABRA2*) have been found to have a link with PTSD [55]. One study found an association of childhood trauma factor score and *GABRA2* SNPs with the risk of developing PTSD in individuals with childhood trauma exposure [56]. In PTSD, RGS2 is a critical protein that plays a role in the process of recovery following trauma, and variations in this gene may be associated with cognitive functioning—specifically with memory and learning processes. A link was also found between the Regulator of G-protein signaling-2 (*RGS2*) rs4606 allele and PTSD under extreme stress conditions [57]. 

**Figure 4:**
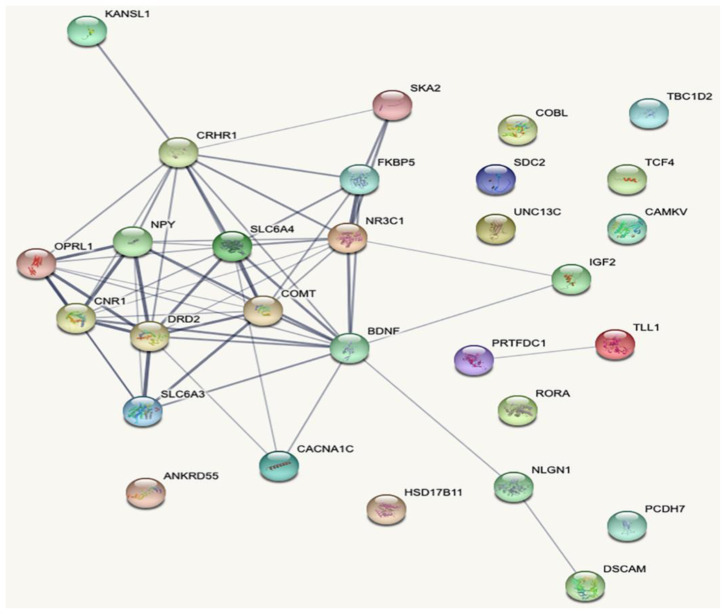
Gene interaction map for PTSD genes generated using string1 webserver. The thickness of the line indicates the strength of the interaction between the genes.

Many GWAS have associated the *RORA* gene with PTSD [58,59]. Confirmatory factor analysis revealed that *RORA* SNP (rs17303244) associated with fear spectrum disorders, such as panic, phobia, and obsessive-compulsive disorder, suggesting that this gene might have a role in fear-related psychopathology [60]. Another GWAS metanalysis in combat-exposed US Marines and sailors identified *PRTFDC1* as a significant PTSD locus [61]. A genome-wide significant association was also reported for SNPs on chromosome 5 in *ANKRD55* with PTSD in African-American samples from the New Soldier Study (NSS) [62]. In a candidate gene association study rs12364283, a functional *DRD2* polymorphism was found to be strongly associated in amphetamine dependent PTSD samples [63]. A recent GWAS identified eight significant loci ( *LINC01360*, *CAMKV*, *KCNIP4*, *HSD17B11*, *MAD1L1*, *SRPK2*, *KANSL1*, *TCF4*) in European-Americans with PTSD re-experiencing symptoms [64]. Genetic studies have reported a link between Apolipoprotein E (APOE) and PTSD. One of the studies found *APOE2* to be associated with increased impaired memory and re-experiencing symptoms in male veterans with combat-related PTSD [65]. A similar study found that mice with human E2 isoform of APOE showed fear extinction impairments with cognitive, behavioral, and neuroendocrine alterations after the occurrence of trauma [66]. Essential genes involved in PTSD and their interactions are shown in Figure 4.

### 3.1. Genetics of Brain Morphology in PTSD

**Figure 5:**
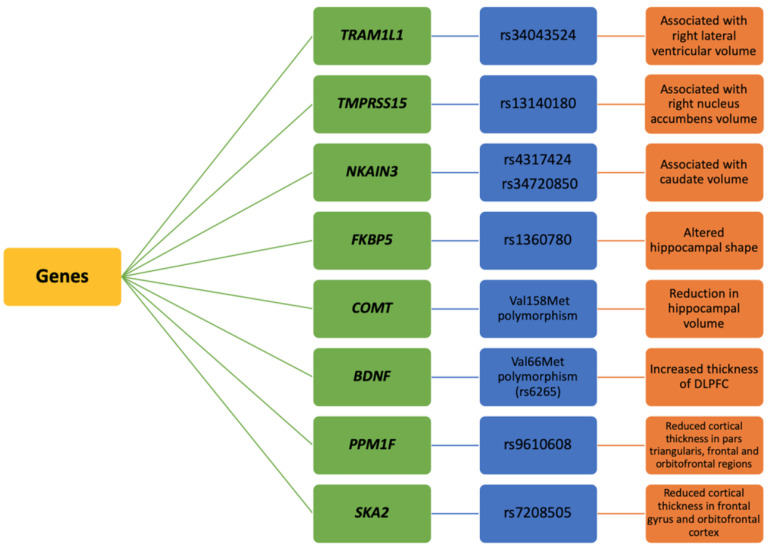
Genes associated with the structural brain changes in PTSD. *TRAM1L1*, *TMPRSS15*, *NKAIN3*, *FKBP5*, *COMT*, *BDNF*, *PPM1F*, and *SKA2* are the most reported genes that affected the brain morphology in PTSD.

Many genes associated with PTSD are found to affect brain morphology (Figure 5). A GWAS in military veteran trauma survivors showed that high linkage disequilibrium (LD) with rs9373240 associated with the right caudate volume, LD with rs34043524, that lies downstream of the *TRAM1L1* gene related to the right lateral ventricular volume [65]. Another study found SNPs in the *TMPRSS15* gene associated with the right nucleus accumbens volume [67,68]. An SNP of *FKBP5* gene (rs1360780) is found to be associated with cingulum connectivity in women with PTSD, and the study reported reduced hippocampal-ACC structural connectivity in PTSD participants compared to traumatized controls [69]. Abnormalities in the cingulum connectivity can affect emotional processes such as fear extinction, a process that is mainly altered in PTSD [70]. A DTI study found lower FA in the left posterior cingulum (PC) in traumatized females who were carriers for two risk alleles of *FKBP5* SNP (rs1360780) as compared to females without risk alleles [5]. Another structural MRI study found an association between *COMT* polymorphism (Val158Met) and hippocampal volume in non-Hispanic war veterans. They found that the participants with high PTSD symptomatology and homozygous for the Val allele showed a greater reduction in the left hippocampal volume compared to the participants with Met allele suggesting that interaction between lower dopamine availability and trauma in Val carriers might negatively contribute to altering the hippocampal structure [71].

Similarly, another study showed the effect of Val158Met polymorphism on ACC volume [6]. They found that the ACC volume between PTSD and non-PTSD individuals was higher in individuals homozygous for the Val allele as compared to the individuals with met allele [6]. A longitudinal MRI study found the increased thickness of the dorsolateral prefrontal cortex (DLPFC) in traumatized South Korean subway disaster survivors as compared to controls. Additionally, the study also found a linear trend of increased DLPFC thickness in trauma-exposed individuals with Val/Val genotype compared to those with methionine genotype [7]. The study also indicated that the Val56Met *BDNF* polymorphism in homozygous Val carriers plays an essential role in the mobilization of DLPFC, and as a result, the chances of recovery in the homozygous Val carriers are higher than the carriers of Met allele [7]. The protein phosphatase gene (*PPM1F*) is an essential negative regulator of cellular pathways related to stress responses and also plays a vital role in serotonergic signaling. An MRI study in non-Hispanic military veterans found the *PPM1F* genotype association with reduced cortical thickness in PTSD individuals. They identified six SNPs in the *PPM1F* gene that associated with PTSD severity and reduced cortical thickness in the right pars triangularis, superior bilateral frontal, and orbitofrontal regions [72]. A study found the *SKA2* gene to be a biomarker for suicide-related psychiatric symptoms and showed that *SKA2* DNA methylation associated with reduced cortical thickness in the superior and inferior frontal gyrus and right orbitofrontal cortex [12].

Brain-derived neurotrophic factor (BDNF) plays a significant role in fear extinction and stress recovery—processes that are disrupted in PTSD. A potential biomarker of PTSD is the BDNF blood level [73]. Hippocampal volume, memory, and susceptibility to PTSD have been reported to be influenced by a single nucleotide polymorphism in *BDNF*, which causes the replacement of valine with methionine at position 66 (Val66Met) [74]. 

Another gene *CACNA1C* encodes for a calcium gated channel and polymorphisms in this gene are found to associate with amygdala volume and activation [75]. The genetic and epigenetic analysis found that an SNP in rs1990322 in *CACNA1C* locus significantly associated with PTSD in highly traumatized police officers [76]. 

### 3.2. Genetics of Neuronal/Functional Changes in PTSD

**Figure 6:**
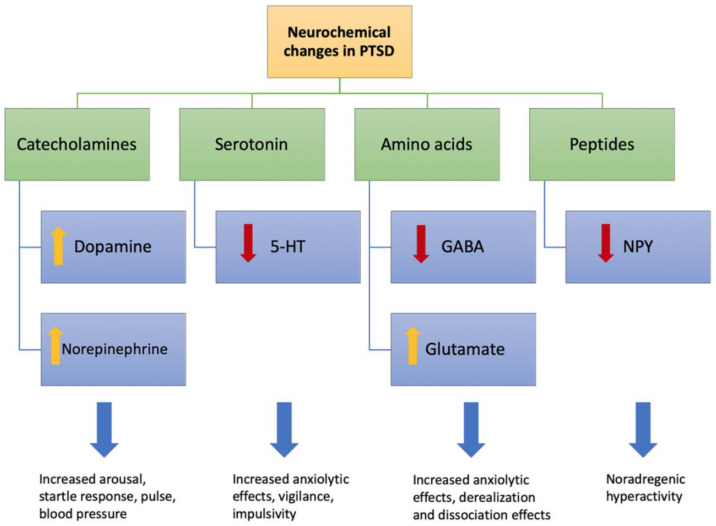
Neurochemical changes associated with PTSD. High levels of dopamine and norepinephrine have been observed in PTSD causing increased blood pressure, anticipation and astonishment response. A low level of serotonin (5HT) in PTSD increases anxiolytic effects. GABA is an important inhibitory neurotransmitter and an alteration of the GABA receptor system results in a decreased level of GABA in PTSD. On the other hand, high levels of glutamate in PTSD can cause excitotoxic effects leading to dissociation phenomena. Also, reduced levels of neuropeptide Y (NPY) in PTSD contributes to noradrenergic hyperactivity.

Neurotransmitters such as dopamine (DA), norepinephrine (NE), serotonin, peptide, amino acid, and opioids play an essential role in regulating stress and fear responses that are shown to be dysregulated in PTSD. In PTSD, high levels of dopamine and norepinephrine have been observed that cause increased blood pressure, arousal, and startle response, while low levels of 5HT cause increased anxiolytic effects. GABA is an important inhibitory neurotransmitter, and in PTSD, there is an alteration of the GABA receptor system. On the other hand, high levels of glutamate can cause excitotoxic effects leading to dissociation phenomena. Also, reduced levels of NPY seen in PTSD contributes to noradrenergic hyperactivity (Figure 6) [77].

Serotonin (5HT) is a monoamine neurotransmitter that is mainly found in the enteric nervous system in the CNS. 5HT modulates stress responses, and it has been shown that 5HT neurons produce anxiogenic effects via 5HT_2_ receptors through projections to the amygdala and hippocampus [78,79]. In PTSD, there is a dysregulation of the brain’s serotonergic systems. Several studies have reported the effects of 5-HTTPLR polymorphism in PTSD [80,81]. There is one long (L) and one short (S) allele in the 5-HTTLPR polymorphism. The expression of the serotonin transporter gene is reduced by the S-allele, ultimately leading to reduced serotonin uptake. A study conducted in returning Iraq and Afghanistan veterans found that veterans carrying the S’ allele of 5-HTTLPR polymorphism were at higher risk of post-deployment adjustment problems [80]. Another study found a higher frequency of G allele of the 5-HT2A promoter region contributing to PTSD susceptibility in a trauma-exposed adult African American population [82]. 

The brain’s dopaminergic system has also been reported to be dysregulated in PTSD. Dopamine is a neurotransmitter that enhances motivation, attention, vigilance, and sleep. The dopamine encoding transporter gene (*SLC6A3*) is present on the chromosome 5p15. An association is found between the 9R (9-repeat) allele of *SLC6A3* and PTSD occurrence [83]. An fMRI study showed that *SLC6A4* SNP rs16965628 modulated activation of the ventrolateral prefrontal cortex while 5-HTTLPR modulated the activation of the left amygdala in a working memory task [9]. Studies showed that individuals who are carriers of the 9R allele had higher chances of developing PTSD, compared to the individuals with the 10R allele [84]. In PTSD, alterations in the levels of a crucial enzyme called Catechol-*O*-methyltransferase (*COMT*) have also been reported [84,85]. *COMT* causes the breakdown of the catecholamine containing neurotransmitters, such as epinephrine, dopamine, and norepinephrine, a key process involved in the regulation of the neurotransmitter system. There is a functional polymorphism in *COMT* at codon 158 involving the rs4680 SNP that causes the substitution of the amino acid valine for methionine. One study found that carriers of the Met158 allele exhibited a decreased ability to overcome conditioned fear, a trait commonly associated with PTSD [86].

Glutamate is a primary neurotransmitter that is involved in every excitation function in the CNS. Glutamate has a major role in synaptic plasticity and is involved in cognitive functions such as learning and memory. Glutamate binds to N-methyl D-aspartate (NMDA) receptors that are mostly found in excitatory synapses. The NMDA receptors are mediators of synaptic transmission, and therefore, they are responsible for the consolidation of trauma memories in PTSD. The neuromodulatory effects of glutamate are mediated by metabotropic glutamate receptors (mGLURs) [87]. A PET imaging reported higher cortical availability of mGLUR5 in PTSD individuals without medication compared to healthy controls. This study also performed postmortem gene expression analysis and found that the expression of mGLUR5 gene did not increase in postmortem tissues of PTSD individuals, suggesting stabilization of mGLUR5 receptors at the surface of neuronal membranes [87]. 

Corticotropin-releasing hormone (CRH) neurons are found in the hypothalamic paraventricular nucleus (PVN) and are a major component of the HPA axis as they integrate stress-related information [88]. It has been found that injecting CRH into the brain of laboratory animals produced stress and anxiety, including neophobia and facilitated fear conditioning [77]. A recent study revealed the absence of *Crh* in the GABAergic neurons by expression mapping in *Crh^CKO–GABA^* mice. This absence of *Crh* from the forebrain GABAergic neurons resulted in social interaction deficits in mice, suggesting the role of CRH in the regulation of social behavior in neuropsychological disorders such as PTSD [89]. The *Crh^CKO–GABA^* mice displayed a resilient phenotype upon exposure to chronic social defeat stress accompanied by a stress-induced expression of *c-fos* and *zif268* genes in several regions of the brain. These studies suggest the significance of GABAergic CRH circuits in the maintenance of social behavior in naïve animals. 

NPY is found to protect against the development of PTSD as it has anxiolytic and stress-relieving properties [90] and plays an important role in homeostatic processes in both the CNS and peripheral nervous system. Lack of NPY promotes maladaptive stress [91] as they are found to inhibit CRH/NE circuits that are involved in stress and fear responses and contribute to the development of PTSD. In yohimbine, which is a noradrenergic α_2_-antagonist study, PTSD patients displayed reduced NPY concentrations in the plasma and blunted NPY responses as compared with controls suggesting that the lack of NPY can contribute to noradrenergic hyperactivity in PTSD [92].

Neurotrophins are an essential family of proteins that are involved in the survival and development of neurons. They are generally known for their nerve growth promoting function and in the regulation of neuronal activity in the central and peripheral nervous systems. BDNF and nerve growth factor (NGF) are essential mediators of synaptic plasticity and neuronal growth and differentiation and play an important role in the pathogenesis of PTSD [93]. 

A study reported several candidate genes involved in synaptic plasticity that might have an essential role in neuronal function in PTSD. They found different polymorphisms in *BDNF*, *CPLX2*, *NTNG1*, *NGF*, *NGFR*, *CHN1*, *FOS*, *JUN*, and *IER5* genes in PTSD patients and controls. According to the study, the rs6265*A allele of the *BDNF* gene, rs4839435*A minor allele of the *NGF* gene, rs734194*T minor allele frequency in *NGFR* gene, and the rs1063169*T minor allele of the *FOS* gene were more frequent in controls as compared to PTSD patients, while the rs1366116*T minor allele of the *CPLX2* gene, rs6330*T allele of the *NGF* gene, the rs6330*T minor allele (CT + TT), and the rs7101*T allele of the FOS gene were more frequent in PTSD patients as compared to the controls [94].

An fMRI study involving a fear processing task in highly traumatized males and females found that SNP (rs6010719) of the *OPRL1* gene associated with increased functional connectivity between the amygdala and posterior insula in PTSD individuals with the GG/GC genotype [11]. Mothers with interpersonal violence-related post-traumatic stress disorder (IPV-PTSD) often have difficulties in parenting due to their frequent exposure to maltreatment and violence. An fMRI study found that *NR3C1* methylation associated with increased parenting stress and decreased prefrontal cortical activity in mothers with IPV-PTSD as compared to healthy controls in response to a video showing a mother and child separation [13]. Another fMRI study showed the association of SNP of the *FKBP5* gene (rs1360780) with hippocampal activation in an African American cohort with interpersonal trauma and post-traumatic symptoms. The study found that carriers of rs1360780 T allele showed increased activation of the hippocampus in an attention bias task and also demonstrated alterations in the hippocampal shape mainly in the CA1 region [4]. In females, PTSD diagnosis and symptoms have been reported to be associated with common variants within the gene encoding adenylyl cyclase-activating polypeptide 1 receptor type I (*ADCYAP1R1)* [95]. A study based on fMRI observed that traumatized women with the CC risk genotype of *ADCYAP1R1* showed higher activity of the amygdala and hippocampus and decreased functional connectivity between the amygdala and hippocampus in response to a threat processing task [10]. Another study found a significant effect of SNP rs406001 in a childhood trauma exposure African American cohort. Although this SNP has no known function, the closest gene found near this SNP is *COBL*, which might have a role in neuronal development. Also, MRI findings showed alteration of white matter regions associated with emotional processing in risk allele carriers [8]

## 4. Diagnostic Model Based on Imaging Genetics in PTSD

At the intersection of genomic medicine and neuroscience, imaging genetics is a rapidly emerging field that combines information from imaging and genetic markers that are implicated in various neurological disorders. The use of structural and functional MRI is increasing worldwide, in part due to its wide range of applications and potential in the diagnosis of neurological illnesses. Different types of brain measures derived from images are now used in the genomic analysis; these not only include volumetric measures from structural MRI but also features from other imaging methods, such as maps based on diffusion that show the connections between brain regions (Table 1). Genetic analyses have also been performed on measures and 3D maps of functional connectivity in the resting state [96,97]. Highly reproducible patterns of task-related and resting-state brain functional connectivity have been found in many functional MRI studies in healthy adults; the reproducibility and heritability of these measures suggest that it may also be possible to discover specific genes that are involved in influencing these traits. The consistency of candidate gene effects associated with neuroimaging is still not satisfactory, as most of the SNP’s that replicate are not in the candidate genes, and the ones that are in the candidate genes have a poor history of replication. Large scale consortia need to be developed to allow efficient replication of genetic effects, and to help in the identification of causal loci and understanding the function of these genes. Advancements in imaging technologies have yielded considerable new insights and data on brain differences associated with behavior and outcomes in various neuropsychiatric disorders [98].

The use of neuroimaging (and in particular, brain MRI) in genetics research can help in the identification of the intermediate phenotypes of PTSD by the characterization of PTSD-related neurobiological traits; genetic analyses of these brain measures can also help in finding associations between alterations in specific sets of genes and the phenotype of the disease. Recently, fMRI studies have been useful in employing paradigms such as emotional stimuli, photographs of facial expressions relevant to PTSD to explore the responses specific to the stimuli in processing circuits linked with basic emotions. Future genetic analyses of these responses show promise in understanding specific genetic variants that may influence brain activation and behavior.

## 5. Potential Challenges and Future Perspective

Albeit huge amounts of data delivered by functional neuroimaging genetics studies on PTSD over time, its neurocircuitry remains incompletely researched and understood. The susceptibility factors that increase the probability of developing PTSD following significant traumatic stress and being identified as biological markers still needs more comprehensive analysis. The translation of these biological markers into clinical practice will require additional measures both technically and statistically. A few studies have reported that different PTSD symptom profiles may have different neural signatures, which could also be challenging to identify a single biomarker linked to PTSD development. Similarly, from the functional imaging studies, it is still unclear that the symptoms of neural activity in response to a specific task is a predisposing risk factor for PTSD or it is a consequence of PTSD. Also, the candidate gene studies in PTSD observed inconsistency between genotypes and neural structure and response patterns that complicate the identification of pure risk genotype in PTSD. These patterns of neural structure and responses are most likely to be influenced by more than one gene, and thus, more genome-wide, and multiple candidate gene studies can help in specifying the genetic associations implicated in PTSD. The approaches described in the above sections related to candidate gene studies and GWAS suffer from the limitation that the genetic neuroimaging studies have not been consistent in terms of their findings, and such inconsistencies suggest the necessity for inclusion of larger sample sizes, diverse demographic clusters, and animal models. Additionally, a major focus of neuroimaging genetics studies has been on the amygdala and hippocampus brain regions, which limit the outcome of such studies, and thus, the inclusion of other brain regions involved in PTSD, such as the prefrontal cortical regions, the anterior cingulate, dorsolateral prefrontal cortex, should be essential targets for future research. As most of the functional neuroimaging and genetic studies have focused on understanding the underlying neurocircuitry and pathobiology of PTSD, rather than developing a diagnostic tool, future studies should focus on advanced technology and data mining to address these limiting factors. Future studies should also consider discovering novel gene targets, and one possible way is to manipulate known genetic targets in animal models in parallel to performing neuroimaging genetics in humans. The other approach towards identification of gene targets and future therapeutics for PTSD will be looking at potential agonists or antagonists and then investigating them in appropriate animal models of fear learning and memory for validation and generation of preliminary evidence. Such studies may be beneficial for the identification of previously unknown genetic targets and novel therapies for PTSD. Future studies can also take advantage of using biomarkers to envisage therapy response in PTSD, and this can be achieved by using individual neurocircuitry profiles to figure out what type of therapy will be very suitable for specific individuals, as various PTSD symptom profiles are actually claimed to have various neural signatures. To determine the origin of functional anomalies, that is whether they are hereditary or develop immediately after the disease appears, could be another direction for future research. The application of statistical innovations, such as genome-wide complex trait analysis and linkage disequilibrium score regression to the current PTSD GWAS data can also help benefit the genomics field in PTSD. Other techniques such as polygenic risk scores and cross-trait linkage disequilibrium score regression can allow researchers to compare the risk of developing PTSD in different phenotypes having different genetic makeup. Further, advancement in the bioinformatics approaches such as gene enrichment and gene pathway analyses can help broaden our understanding of the pathophysiology of PTSD. The application of integrative omics that includes the genome, metabolome, transcriptome, epigenome, and microbiome will help to develop computational technologies that can help in the discovery and identification of various molecular targets and diagnostic biomarkers relevant to PTSD. This multi-omics approach will also facilitate the identification of novel drug targets and automated processes such as high throughput screening for the discovery of new drugs. In addition, advancement in technologies can help in searching for novel blood-based biomarkers that could be more valuable for individuals who are potentially more exposed to traumatic stressors or situations, such as warzone veterans, marines, police, and emergency medical teams. 

To sum up, it is of utmost importance to emphasize that neuroimaging genetics is a relatively new, promising, and fast-evolving research subject. The collaborative research, multimodal approach and better integration of neuroimaging observations from the past will considerably enhance our understanding of the underlying cause of symptoms that show up in PTSD, and how genetics contributes to phenotypic neural differences, which together may prove extremely effective in the treatment and recovery in PTSD patients.

## 6. Conclusions

Within the last few years, there has been significant progress in the diagnosis and understanding of PTSD, assisted by neuroimaging techniques such as MRI. With advances in the field of molecular biology—as well as imaging technologies—there has been an in-depth investigation of the molecular and neural mechanisms underlying PTSD. Dysregulated molecular pathways underlying PTSD are also involved in response to fear and stress; the effects of these molecular pathways on the structure and function of neural systems can be studied by combining genomic analyses with neuroimaging techniques. The combination of all these techniques and the strategy for interpreting and translating the data acquired may provide an opportunity to develop various diagnostic tests for PTSD that may further enhance the diagnosis and clinical management of this illness.

## Figures and Tables

**Table 1:** Neuroimaging-genetics studies in PTSD.

Gene (s)	Risk Allele/Polymorphism	Subjects	Method Used	Affected Brain Regions	Findings	References
*FKBP5*	rs1360780	Healthy adults and risk allele carriers for PTSD	fMRI (Attention bias task)	Hippocampus	T risk allele carriers showed increased hippocampal activation and altered hippocampal shape	[4]
	rs1360780	Traumatized females	DTI, FA	Entorhinal cortex	Lower FA in the left posterior cingulum of risk allele carriers	[5]
*COMT*	Val158Met	PTSD individuals and healthy adults	MRI	ACC	PTSD-positive participants that were Met carriers showed increased right ACC volume	[6]
*BDNF*	Val66Met polymorphism (rs6265)	Psychologically traumatized disaster survivors and healthy controls	MRI	Prefrontal cortex	Increased DLPFC thickness in the trauma-exposed individuals	[7]
*COBL*	rs406001	PTSD individuals	sMRI	Prefrontal cortex, amygdala	Alterations in white matter integrity in brain regions associated with emotional processing in affected individuals	[8]
*SLC6A4*	rs16965628 5-HTTLPR	Post-9/11 veterans with PTSD and trauma-exposed controls	fMRI (working memory task)	Amygdala	rs16965628 SNP modulated task-related ventrolateral PFC activation in patients with PTSD. 5-HTTLPR modulated left amygdala activation during the working memory delay period in S allele carriers with PTSD	[9]
*ADCYAP1R1*	rs2267735	Highly traumatized cohort of women	fMRI (threat-processing task)	Amygdala and hippocampus	Increased responses to fearful stimuli in the amygdala and hippocampus of risk allele carriers (CC)	[10]
*OPRL1*	rs6010719	Highly traumatized males and females	fMRI (fear-processing task)	Amygdala	risk allele carriers (GG/GC) showed increased functional connectivity between amygdala and posterior insula as compared to the CC genotype	[11]
*SKA2*	rs7208505	Trauma-exposed veterans	sMRI	Prefrontal cortex	*SKA2* methylation associated with reduced cortical thickness in prefrontal cortex in trauma-exposed veterans	[12]
*NR3C1*		Mothers with interpersonal trauma	fMRI (mother-child interaction sequences of free-play and separation)	Prefrontal cortex	maternal mPFC activity correlated positively to *NR3C1* methylation and negatively to parenting stress and maternal IPV-PTSD in response to a video showing mother and child separation	[13]
